# Years of sunlight exposure and cataract: a case-control study in a Mediterranean population

**DOI:** 10.1186/1471-2415-7-18

**Published:** 2007-11-26

**Authors:** María Pastor-Valero, Astrid E Fletcher, Bianca L de Stavola, Vicente Chaqués-Alepúz

**Affiliations:** 1 London School of Hygiene and Tropical Medicine, London, UK; 2 Centro de Especialidades de Burjassot, Valencia, Spain

## Abstract

**Background:**

We aimed to investigate the relation between sunlight exposure and risk of cataract.

**Methods:**

We carried out a frequency-matched case-control study of 343 cases and 334 controls attending an ophthalmology outpatient clinic at a primary health-care center in a small town near Valencia, Spain.

All cases were diagnosed as having a cataract in at least one eye based on the Lens Opacification Classification system (LOCS II). Controls had no opacities in either eye. All cases and controls were interviewed for information on outdoor exposure, "usual" diet, history of severe episodes of diarrhea illness, life-style factors and medical and socio-demographic variables. Blood antioxidant vitamin levels were also analyzed. We used logistic regression models to estimate sex and age-adjusted odds ratios (ORs) by quintiles of years of occupational outdoor exposure, adjusting for potential confounders such as smoking, alcohol consumption, serum antioxidants and education.

**Results:**

No association was found between years of outdoor exposure and risk of cataract. However, exploratory analyses suggested a positive association between years of outdoor exposure at younger ages and risk of nuclear cataract later in life.

**Conclusion:**

Our study does not support an association with cataract and sunlight exposure over adult life.

## Background

Laboratory studies demonstrate that exposure to ultraviolet radiation (UVR) induces lens opacification [1-5]. In human populations, the strongest evidence is provided by studies of an occupational group with high outdoor exposure where ocular UVR has been estimated [6,7]. Among the general population exposure to sunlight has been related more frequently to cortical cataracts [6,8-10] but not observed in all [11] or else observed in men but not in women [12]. Only two studies have found an association between sunlight exposure at younger ages and nuclear cataracts in adult life [13,14]. A few epidemiological studies have been conducted in European populations. A hospital-based case-control study conducted in Parma, Northern Italy, showed increasing risk of cortical cataracts with a 4-level scale of the estimated time spent outdoors[8]. In a small population-based study in the north of Finland, "working outdoors" was a risk factor for cortical cataracts in women but not in men [15]. A French study (The POLA study), in the south of France, showed an association of annual ambient solar radiation with cortical and mixed cataracts (mainly cortical and nuclear) and a weak trend for nuclear cataracts [16].

We undertook a case-control study in Valencia province, a coastal region of Spain, where sunshine levels are well above the European average [17]. Our overall aim was to examine the associations between sunlight exposure and intake of antioxidants with cataract. In this paper, we report the association for sunlight exposure.

## Methods

### Study Population

We carried out a frequency-matched case-control study of 343 cases and 334 controls to investigate potential risk factors for cataract which included years of outdoor exposure and intake of antioxidant micro- nutrients. Detailed information on the methodology and the nutritional results has been described previously [18].

The study was conducted from July 1994 to September 1995 in the city of Burjassot, on the Mediterranean east-coast of Spain, within the province of Valencia. All cases and controls were recruited among patients ages 55 to 74 attending the ophthalmology outpatient day-care clinic at the town's primary health-care center (Centro de Especialidades de Burjassot, hereafter referred to as CEB). The CEB belongs to the National Health Care System and is a primary referral health care centre for all individuals living in the same geographical area. The majority of the population in the Valencia province uses the National Health Care System as the main medical service for cataract problems.

A case consisted of any patient from 55 to 74 years of age who was diagnosed with nuclear, cortical, posterior subcapsular, or mixed cataract (i.e. any combination of the types listed) in at least one eye, and of grade ≥ 1 for cortical and posterior subcapsular cataract and ≥ 2 for nuclear opacities, according to the LOCS system Version II (Lens Opacification Classification System II) [19]. The lenses were assessed by slit lamp examination after pupil dilatation and graded using the grading scale for opalescence from 0 to III for nuclear cataract, from 0 to IV for cortical cataract, and from 0 to III for posterior sub capsular cataract. Grade 0 refers to no opacities for any of the subtypes.

Each participant's lens status was independently classified by two observers, and agreement reached for every diagnosis.

If the patient was aphakic or pseudophakic in one eye, he/she could still be a case if the other eye was diagnosed as having any type of cataract. Controls consisted of other patients not affected by cataracts and frequency-matched by age (within 5 years) and gender with the cases. Among these patients, both eyes had no lens opacities, according to the LOCS II classification system. Participants with unilateral congenital or traumatic cataract were classified according to their other eye. We excluded patients if they (i) were on special diets; (ii) had an intra-ocular pressure of ≥ 21 mm Hg; or (iii) had conditions or medical treatments known to be associated with an increased risk of cataract (including people with diabetes). Diabetics were excluded for two main reasons (i) Diabetics undergo regular ophthalmologic examinations for risk of diabetic retinopathy. Surveillance bias therefore may make diabetic patients more likely to be diagnosed with cataract at an earlier stage than the general population from which the cases were drawn (ii) diabetics follow special diets. Cases were also excluded if they had a congenital or traumatic cataract or had a cataract associated with exposure to radiation, or toxic agents.

The study design adhered to the tenets of the Declaration of Helsinki and was approved by the Ethical Committees of the London School of Hygiene and Tropical Medicine, England, and the CEB, Spain. Written informed consent was obtained from each participant.

### Interview data

Data were collected by trained study interviewers from the Valencia Institute of Public Health (IVESP) who were unaware of cataract status and the hypotheses under study. The data collected included measurements of height and weight, as well as a structured questionnaire for information on socio-demographic variables, history of severe episodes of diarrhoeal illness; all medications currently being used, "usual" diet using a validated Food Frequency questionnaire; current and past tobacco and alcohol consumption; use of vitamin supplements; and information on outdoor exposure (see below). Blood antioxidant vitamin levels were also analyzed [18].

### Measurement of Sunlight Exposure

Years of outdoor exposure were estimated according to a modified version of the empirical model developed by Rosenthal et al. [20]. The original Rosenthal exposure model uses three data sources (i) a questionnaire to collect personal lifetime outdoor exposure; (ii) UVR meteorological data (average annual ultraviolet radiation flux, reflectiveness of terrains); and (iii) laboratory data on ocular protection provided by hats, glasses and sunglasses.

We used a questionnaire designed for use among the general population and provided by Dr S. K. West in 1993 (Dana Center for Preventive Ophthalmology, Johns Hopkins University, USA). This was slightly modified to include some additional information (e.g. season of exposure). The questionnaire's main component was the chronological history of time spent outdoors between 10 AM to 4 PM (when UVR is at its maximum in the Northern Hemisphere), during working periods from age 25 to the date of interview (or diagnosis). Eighty percent of all cases were newly diagnosed at the time of the interview, but 20% had a clinic record of a diagnosis of cataract within the recruitment period (1994 to 1995). For these, information on sunlight exposure was collected up to the date of diagnosis.

Periods during retirement or spent working as a housewife were considered as employment periods. We also collected information on time spent outdoors on weekends during the summer but did not use it because it was found to be inconsistently reported by people in different occupations. The questionnaire assumed that everybody had 2 days per week-end. However, for the most frequent occupations among the participants such as farmers, bricklayers, and shop assistants, the usual work week was 6 days; and for others, such as housewives and retired people, 7 days. For people who worked more than 5 days and reported leisure time activities it was not possible to establish how much of the daily exposure over the week-end was due to work or leisure activities and therefore there was a possibility of double counting the hours of sunlight exposure. The outdoor exposure information was collected for each occupation, with winter (October to March) and summer (April to September) periods separated. For those who were working in shifts, each shift was considered a separate job, since exposure to sunlight might vary considerably depending on the job schedule. We did not have information on local meteorological data regarding radiant UV-B for the period under investigation. Instead, we used the average monthly number of sunlight hours in Valencia from 1938 to 1990, covering most of the exposure period for the study participants [17], to account for differences in summer and winter exposures. This led to weighing winter years of exposure by a factor of 0.39, and the summer ones by a factor of 0.61. We separately calculated the total number of years of winter and summer exposure for every job period and then added the contributions from all of them. The weighted average of the winter and summer years of exposure thus represents the cumulative number of years of outdoor exposure between 10 AM and 4 PM from age 25 to the date of interview or diagnosis.

Shown below is the model for determining years of outdoor exposure (OE).

*OE *= {0.39 *Σ*_pw _*years*_pw _[*hrs*_pw _*x days*_pw_] + 0.61 *Σ*_*qs *_*years*_*ps *_[*hrs*_qs _*x days*_qs_]}/(365.25*24)

Where

*pw *= Indicator for summer employment periods *p*

*q s *= Indicator for winter employment periods *q*

*years*_*pw *_= Total number of years in employment period *pw*

*years*_*qs *_= Total number of years in employment period *qs*

days_pw_= Number of weekdays per year in employment period *pw*

*days*_*qs*_= Number of weekdays per year in employment period *qs*

*hrs*_*pw *_= Number of hours per day between 10 AM and 4 PM spent outdoors during employment period *pw*

*hrs*_*qs *_= Number of hours per day between 10 AM and 4 PM spent outdoors during employment period *qs*

Finally, data on the use of protective devices such as hats, spectacles, and sunglasses while outdoors was collected and was included in an alternative definition of years of outdoor exposure, hereafter referred to as "corrected years of outdoor exposure".

The responses for the use of protective devices ranged from "never," "less than half the time," "half the time," and "more than half the time," to "all the time." This measure weighed the number of hours spent outdoors during a given employment period by 1, provided no protective factor was used, or by a term dependent on which type of protection was used. We used the same weights as those arrived at by Rosenthal *et al*. in studies of the ocular dose of UV radiation from exposure to sunlight [21-23]. According to their results, the level of ocular protection provided by spectacles is 79%, i.e. the percentage of time that a person wears spectacles while outdoors is multiplied by a factor of 0.21. The protection provided by the use of sunglasses is 93% (hence the factor is 0.07); and that provided by hats an average of 47% (ranging from 75% for a wide-brimmed hat and 20% for a short-brimmed-hat).

### Statistical Analysis

The analyses were first carried out taking cataract as a general outcome (all types of cataracts combined), and then, repeated by the specific type of cataract to explore whether the exposures had any different effect by type of cataract. The two measures of exposure, outdoor years and corrected outdoor years, were categorized into fifths according to the quintiles of the controls distribution, with the lowest category treated as the reference group. The associations between the categories of these two exposure variables and risk of cataract were evaluated in terms of odds ratios (ORs) with 95% confidence intervals (95% CI), using two separate age-and sex-adjusted logistic regression models.

We examined the effects of potential confounders, namely dietary intake; plasma antioxidant levels; episodes of severe diarrheal illness; supplementary use of antioxidant vitamins; body mass index; regular use of aspirin, anti-hypertensive and anti-gout medication by including these factors sequentially into the two separate logistic regression models and examining whether they changed or modified the original ORs. Education, "pack-years" of cigarette consumption and alcohol history were forced into all models. Final models used multiple logistic regressions to estimate odds ratios adjusted by smoking history, alcohol consumption, and education and serum levels of ascorbic acid, retinol and lycopene. Overall significance and linear trends were assessed using Likelihood Ratio Tests (LRT) [24].

To explore whether either measure of years of outdoor exposure were relevant for a particular type of cataract, the two models were also fitted using all the controls and either case subset with pure nuclear, pure posterior, or pure cortical. Participants were classified as having a "pure" type of cataract when only one type of lens opacity was present in one or in both eyes. All statistical analyses were performed using *Stata 6 *software [25].

## Results

We recruited a total of 347 cases and 345 controls, frequency-matched by sex and age (± 5 years). There were no refusals from anyone but 4 cases and 11 controls did not attend the interviews or blood-collection session, and therefore were excluded from this study.

Table 1 shows the distribution of specific socio-demographic characteristics of the cases and controls included in the study. The mean age was 66.3 years (median = 67) for the cases, and 66.4 years (median = 67) for the controls.

**Table 1:** Distribution of socio-demographic characteristics of cases and controls

	**Cases**		**Controls**	
		
**Variables**	**n**	**%**	**n**	**%**
**All**	343	100.0	334	100.0
				
**Gender:**				
				
Male	149	43.4	133	39.8
Female	193	56.3	201	60.2
Unknown	1	0.3	0	0.0
				
**Age group ***(years***):**				
55–59	40	11.7	40	12.0
60–64	82	23.9	84	25.1
65–69	106	30.9	98	29.3
70–74	114	33.2	112	33.6
Unknown	1	0.3	0	0.0
				
**Marital status:**				
				
Single	21	6.1	13	3.9
Married/in couple	252	73.5	253	75.8
Widowed	63	18.4	63	18.8
Divorced	7	2.0	5	1.5
Unknown	0	0	0	0
				
**Occupational situation:**				
				
Full/part time employed	18	5.2	19	5.7
Unemployed	12	3.5	11	3.3
Housewife	168	49.0	174	52.1
Retired/others	143	41.7	130	38.9
Unknown	2	0.6	0	0.0
				
**Education**				
				
Illiterate	80	23.4	52	15.6
Incomplete or complete primary school	213	62.0	204	61.1
Secondary/junior high school	40	11.7	55	16.4
Senior high school and University	10	2.9	23	6.9

Table 2 shows the frequency distribution of type of cataract diagnosed among the study subjects. The most frequent type of cataract was pure nuclear (N = 100, 30.0%) followed by pure posterior (N = 62, 18.9%) and pure cortical (N = 40, 12.0%).

**Table 2:** Distribution of cases by type of cataract

**Type of cataract**	**N**	**%**
**All**	**343**	**100.0**
		
**Pure:**	**202**	**60.96**
		
- Nuclear	100	30.03
- Cortical	40	12.02
- Posterior	62	18.91
		
**Mixed:**	**141**	**39.03**
		
- Nuclear-cortical	33	9.13
- Nuclear-posterior	45	12.46
- Posterior-cortical	63	17.44

Table 3 shows the results of two separate regression models between the variables for years of outdoor exposure (corrected and non-corrected outdoor exposure) and risk of cataract. No association or trend was found between years of outdoor exposure and risk of cataract. Alcohol and pack-years of cigarette consumption were not found to be confounders for years of outdoor exposure. However, education was found to have a mildly confounding effect for cataract. Education was independently associated with cataract; those completing secondary education and above had lower odds ratios compared to those who were illiterate (OR = 0.26; 95%CI = 0.11–0.60).

**Table 3:** Odds ratios (OR) and 95% confidence intervals (CI) of cataract (all types) for categories of corrected and non-corrected outdoor exposure ^¶^

	**Outdoor exposure from 10 am-4 pm corrected for protective factors (years; fifths)**					
	< 0.073*	**0.073 – 0.190**	**0.191 – 0.428**	**0.429 – 0.800**	**> 0.800**	***Trend test (p-value)*^‡^**
	
No. of cases = 332	72	56	74	59	71	
No. of controls = 327	66	65	66	65	65	
						
OR†	1	0.80	1.06	0.84	0.99	*0.98*
(95% CI)		(0.49, 1.31)	(0.65, 1.71)	(0.51, 1.37)	(0.61, 1.61)	
						
OR**§**	1	0.97	1.03	0.84	0.99	*0.81*
(95% CI)		(0.56, 1.68)	(0.60, 1.76)	(0.48, 1.45)	(0.57, 1.73)	
						
	**Outdoor exposure from 10 am-4 pm (years; fifths)**					
	< 0.312*	**0.312 – 0.612**	**0.613 – 0.968**	**0.969 – 1.428**	**> 1.428**	***Trend test (p-value)*^‡^**
	
No. of cases = 332	73	57	65	43	94	
No. of controls = 327	66	65	66	65	65	
						
OR†	1	0.81	0.91	0.58	1.20	*0.49*
(95% CI)		(0.47, 1.38)	(0.53, 1.55)	(0.32, 1.02)	(0.71, 2.04)	
						
OR**§**	1	0.77	0.85	0.57	1.19	*0.83*
(95% CI)		(0.45, 1.31)	(0.50, 1.47)	(0.32, 1.03)	(0.69, 2.03)	

^¶ ^A total of 11 cases and 7 controls were excluded because of missing values

* Reference category

^‡ ^Chi-squared test for linear trend with 1 df.

† ORs are adjusted for sex and age.

^§ ^ORs are adjusted for sex, age, smoking history, alcohol consumption, education and serum levels of antioxidants

Once we examined the relation between years of outdoor exposure and each separate type of cataract we found increased odds with increasing exposure for pure nuclear cataract, but decreases in odds for the others (Table 4). Alcohol and pack-years of cigarette consumption were not found to be confounders for years of outdoor exposure and for any type of pure cataract. Analyses by type of cataract also found that lower levels of education were associated with all types of cataract (results not shown). However, no confounding effects or interactions were found between years of outdoor exposure and the blood levels of these antioxidants.

**Table 4:** Odds ratios (OR) and 95% confidence intervals (CI) of cataract subtypes for categories of corrected and non-corrected outdoor exposure ^¶^

	**Outdoor exposure from 10 am-4 pm corrected for protective factors (years; fifths)**					
	< 0.073*	**0.073 – 0.190**	**0.191 – 0.428**	**0.429 – 0.800**	**> 0.800**	***Trend test (p-value)*^‡^**
	
**Pure nuclear (No. of cases = 100)**						
OR**§**	1	2.37	3.36	2.02	3.19	*0.05*
(95% CI)		(0.88, 6.38)	(1.32, 8.54)	(0.75, 5.44)	(1.24, 8.21)	
**Pure cortical (No. of cases = 40)**						
OR**§**	1	0.55	0.58	0.51	0.29	*0.09*
(95% CI)		(0.18, 1.71)	(0.20, 1.72)	(0.16, 1.67)	(0.78, 1.09)	
**Pure posterior (No. of cases = 62)**						
OR**§**	1	0.76	1.01	0.58	0.57	*0.23*
(95% CI)		(0.27, 2.12)	(0.40, 2.56)	(0.21, 1.62)	(0.20, 1.60)	
						
	**Outdoor exposure from 10 am-4 pm (years; fifths)**					
	< 0.312*	**0.312 – 0.612**	**0.613 – 0.968**	**0.969 – 1.428**	**> 1.428**	***Trend test (p-value)***
	
**Pure nuclear (No. of cases = 100)**						
OR**§**	1	2.22	1.28	1.86	3.68	*0.01*
(95% CI)		(0.88, 5.61)	(0.47, 3.52)	(0.70, 4.91)	(1.50, 9.01)	
**Pure cortical (No. of cases = 40)**						
OR**§**	1	0.50	0.47	0.12	0.67	*0.28*
(95% CI)		(0.17, 1.44)	(0.16, 1.42)	(0.03, 0.55)	(0.22, 2.00)	
**Pure posterior (No. of cases = 62)**						
OR**§**	1	0.42	0.62	0.42	0.57	*0.09*
(95% CI)		(0.15, 1.16)	(0.24, 1.60)	(0.15, 1.14)	(0.22, 1.45)	

A total of 7 controls were excluded because of missing values

* Reference category

^‡ ^Chi-squared test for linear trend with 1 df.

§ORs are adjusted for sex, age, smoking history, alcohol consumption, education and serum levels of antioxidant vitamins

Our study was not designed to analyze the association between sunlight exposure by type of cataract, and thus, the results reported here are only explorative and should be interpreted with caution. In this context, we also examined the relation between exposure to sunlight during two 20-year periods: young adult life (ages 25–45), and older adult life (ages 46–64) and risk of pure nuclear cataract. Early outdoor years of exposure above 1.59 (fifth quintile during younger years), showed increased odds (OR = 3.05; 95% CI = 1.25–7.42). Later sunlight exposures above 1.80 (fifth quintile during older years) showed no increased odds, (OR = 1.01; 95% CI = 0.39–2.60).

## Discussion

Our results did not show an association between occupational sunlight exposure when all types of cataract were combined making them similar to the findings of other studies [11,26-30]. When examining the risk of cataract separately by type, we found an association between occupational years of outdoor exposure and nuclear cataract. In particular, uncorrected outdoor exposures above 1.42 years (i.e. the top fifth) showed an increase in odds, greater than threefold (OR = 3.68; 95% CI = 1.50–9.01). Similarly, corrected exposure above 0.80 years (i.e. the top fifth) was found to increase the odds by a factor of about 3 (OR = 3.19; 95% CI = 1.24–8.21).

Our results are not consistent with an association of sunlight exposure and cortical cataract [6,8-10,12,16,29] or with the negative association with nuclear cataract reported by some [6,8,9,11,12,26,27,29]

We are only aware of three studies that have suggested an association between sunlight exposure and nuclear cataract. A French study found a weak association of this type in a coastal Mediterranean population, in some respects similar to ours [16]. The methodology used in the French-study to measure UVR exposure was comparable to our study but included meteorological data on ambient solar radiation. Although we did not have this information we think it unlikely that it would have altered our results in any significant way. Most our participants (97%) lived all their entire life in Valencia and therefore are not expected to show much heterogeneity in UVR exposure other than that provided by occupational and leisure activities.

In Nambour, Australia, the presence of nuclear cataract later in life was strongly associated with occupational sun exposure between ages 20 and 29 (OR = 5.9; 95% CI = 2.1–17.1). The authors reported that the strength of the association between sun exposure during the fourth and subsequent decades of life and cataracts was greatly reduced after adjusting for sun exposure between ages 20 and 29 [13]. In a Japanese study, lifetime UVB exposure was investigated in relation to type of lens opacities. Lifetime cumulative UVB exposure and exposure after the teenage years correlated with the presence of nuclear opacities later in life in females [14].

Obtaining accurate measurements of personal past sunlight exposure is not a trivial task. One limitation of our study lies in the design of the questionnaire itself, particularly, when used to collect information from certain specific groups of participants. Recall was particularly difficult for those who did not have a type of work defined by a fixed schedule, for example housewives. The majority of the women participating in this study were housewives (87%), and most had been housewives all their life. However, some evidence of the validity of our questionnaire was provided by the positive association between levels of education and type of occupation, and the measures of years of outdoor exposure. For instance, farmers showed the highest median sunlight exposure (n = 86, median = 2.10 years), while people working indoors such as receptionists or shop assistants had the lowest (n = 42, median = 0.89 years). Further, higher educational level was inversely associated with years of exposure to sunlight: illiterate people had the highest median exposures (n = 132, median = 2.0 years) while those who went to high school or university had the lowest (n = 33; median = 0.93 years).

Factoring in ocular protection factors did not alter the original results. In general, the practice of wearing protective devices was quite similar between the cases and the controls. Thus, 52% of our cases, as compared to 48% of our controls, reported having ever worn spectacles, 53% of cases and 47% of controls, a hat, and 47% of cases and 53% of controls, sunglasses. Several studies have shown that wearing a hat or sunglasses reduces the amount of light entering the eye [21-23]. However, in Spain, there is no medical advisory from either ophthalmologists or public-education campaigns indicating the possibility that sunlight could contribute to the onset of cataracts. If there were a misclassification of years of outdoor exposure, there would be no reason to believe that this would be greater in people with cataracts. Inaccuracy in reporting is likely to have similarly occurred in both cases and controls, leading to a reduction in the possible magnitude of the odds ratio.

The association between sunlight exposure and nuclear cataract is difficult to explain solely on the basis of direct absorption, since the damage would be expected to appear in the cortical area first, where most of the UV rays reaching the lens would be absorbed by UV filters [31,32]. A combination of internal (generated within the nucleus) and external oxidative factors (outside the nucleus), with aging being by far the major risk [32], might contribute to the aetiology of nuclear cataract. The lens is a tissue that maintains every fiber cell ever formed and continues to grow throughout life with the old fibers compressed into the center of the nucleus and the cortical area composed of the newly formed fibers [31].

We found that early occupational exposure to sunlight, from 25 to 45 years of age, increased the risk of nuclear cataract later in life. UV filters concentrations decreases at approximately 12% per decade [32]. It may be that lifetime cumulative sunlight exposure in conjunction with internal factors, e.g. the development of an internal barrier in middle age, may play an important role in the development of nuclear cataract in adult life. Our findings showed no increased risk of cortical cataract as a result of outdoor exposure. Another possibility involves dietary vitamin C intake, which among our study population was high (157 mg/day) [18]. This high intake has also been observed in another Spanish population from the same geographical area [33] and exceeds the intakes of other populations [34-36]. High vitamin C intake might especially have protected the cortical area of the lens from UVR oxidative stress. Indeed, our antioxidant analyses showed that plasma ascorbate levels greater than 49 μmol/L were associated with a 64% reduction in the risk of cataracts and this protective effect was consistent across all types [18].

We did not find evidence for any interaction between levels of outdoor exposure and antioxidant intake, but the power of our study to detect such interactions was very low. Finally, the possibility of residual confounding and chance are alternative explanations for our findings. The small numbers of cortical (40) and posterior (62) cases could have increased the risk for Type II error, though less so for nuclear cases (100).

## Conclusion

We found no association with cataract and occupational sunlight exposure when measured over adult lifetime. Sunlight exposure at younger ages might be a risk factor for nuclear cataract but the observed association needs confirmation by future studies which specifically analyze early exposure to sunlight.

## Competing interests

The authors declare that they have no competing interests.

## Authors' contributions

MPV graded cataract, designed the study, analyzed the data and wrote the first draft of the manuscript.

AEF designed the study and critically reviewed and modified the manuscript.

BLS directed the statistical analyses and critically reviewed and modified the manuscript.

VCA graded the cataract and critically reviewed and modified the manuscript.

All authors reviewed and modified the manuscript. All authors read and approved the final manuscript.

## Pre-publication history

The pre-publication history for this paper can be accessed here:


